# The effects of online game addiction on reduced academic achievement motivation among Chinese college students: the mediating role of learning engagement

**DOI:** 10.3389/fpsyg.2023.1185353

**Published:** 2023-07-13

**Authors:** Rui-Qi Sun, Guo-Fang Sun, Jian-Hong Ye

**Affiliations:** ^1^BinZhou College of Science and Technology, Binzhou, China; ^2^Binzhou Polytechnic, Binzhou, China; ^3^Faculty of Education, Beijing Normal University, Beijing, China; ^4^National Institute of Vocational Education, Beijing Normal University, Beijing, China

**Keywords:** college students, online game addiction, learning engagement, reduced academic achievement motivation, online games

## Abstract

**Introduction:**

The present study aimed to examine the effects of online game addiction on reduced academic achievement motivation, and the mediating role of learning engagement among Chinese college students to investigate the relationships between the three variables.

**Methods:**

The study used convenience sampling to recruit Chinese university students to participate voluntarily. A total of 443 valid questionnaires were collected through the Questionnaire Star application. The average age of the participants was 18.77 years old, with 157 males and 286 females. Statistical analysis was conducted using SPSS and AMOS.

**Results:**

(1) Chinese college students’ online game addiction negatively affected their behavioral, emotional, and cognitive engagement (the three dimensions of learning engagement); (2) behavioral, emotional, and cognitive engagement negatively affected their reduced academic achievement motivation; (3) learning engagement mediated the relationship between online game addiction and reduced academic achievement motivation.

## 1. Introduction

Online games, along with improvements in technology, have entered the daily life of college students through the popularity of computers, smartphones, PSPs (PlayStation Portable), and other gaming devices. Online game addiction has recently become a critical problem affecting college students’ studies and lives. As early as 2018, online game addiction was officially included in the category of “addictive mental disorders” by the World Health Organization (WHO), and the International Classification of Diseases (ICD) was updated specifically to include the category of “Internet Gaming Disorder” (IGD). Prior research investigating Chinese college students’ online game addiction status mostly comprised regional small-scale studies. For example, a study on 394 college students in Chengde City, Hebei province, China showed that the rate of online game addiction was about 9% (Cui et al., 2021). According to the results of an online game survey conducted by China Youth Network (2019) on 682 Chinese college students who played online games, nearly 60% of participants played games for more than 1 h a day, over 30% stayed up late because of playing games, over 40% thought that playing games had affected their physical health, over 70% claimed that games did not affect their studies, and over 60% had spent money on online games. This phenomenon has been exacerbated by the fact that smartphones and various portable gaming devices have become new vehicles for gaming with the development of technology. The increase in the frequency or time spent on daily gaming among adolescents implies a growth in the probability of gaming addiction, while an increase in the level of education decreases the probability of gaming addiction (Esposito et al., 2020; Kesici, 2020). Moreover, during the COVID-19 pandemic, adolescents’ video game use and the severity of online gaming disorders increased significantly (Teng et al., 2021).

A large body of literature on the relationship between problematic smartphone use and academic performance has illustrated the varying adverse effects of excessive smartphone obsession (Durak, 2018; Mendoza et al., 2018; Rozgonjuk et al., 2018). These effects are manifested in three critical ways: first, the more frequently cell phones are used during study, the greater the negative impact on academic performance and achievement; second, students are required to master the basic skills and cognitive abilities to succeed academically, which are negatively affected by excessive cell phone use and addiction (Sunday et al., 2021); third, online game addiction negatively affects students’ learning motivation (Demir and Kutlu, 2018; Eliyani and Sari, 2021). However, there is currently a lack of scientifically objective means of effective data collection regarding online game addiction among college students in China, such as big data. Hong R. Z. et al. (2021) and Nong et al. (2023) suggested that the impact of addiction on students’ learning should be explored more deeply.

Since the 1990s, learning engagement has been regarded as a positive behavioral practice in learning in Europe and the United States, and plays an important role in the field of higher education research (Axelson and Flick, 2010). Recently, studies on learning engagement among college students have also been a hot topic in various countries (Guo et al., 2021). According to Fredricks et al. (2004), learning engagement includes three dimensions: behavioral, emotional, and cognitive.

The concept of behavioral engagement encompasses three aspects: first, positive behavior in the classroom, such as following school rules and regulations and classroom norms; second, engagement in learning; and third, active participation in school activities (Finn et al., 1995). Emotional engagement refers to students’ responses to their academic content and learning environment. The emotional responses to academic content include students’ emotional responses such as interest or disinterest in learning during academic activities (Kahu and Nelson, 2018), while the emotional responses to the learning environment refer to students’ identification with their peers, teachers, and the school environment (Stipek, 2002). Cognitive engagement is often associated with internal processes such as deep processing, using cognitive strategies, self-regulation, investment in learning, the ability to think reflectively, and making connections in daily life (Khan et al., 2017). Cognitive engagement emphasizes the student’s investment in learning and self-regulation or strategies.

According to Yang X. et al. (2021), learning engagement refers to students’ socialization, behavioral intensity, affective qualities, and use of cognitive strategies in performing learning activities. Besides, Kuh et al. (2007) argued that learning engagement was “the amount of time and effort students devote to instructional goals and meaningful educational practices.” Learning engagement is not only an important indicator of students’ learning process, but also a significant predictor of students’ academic achievement (Zhang, 2012). It is also an essential factor in promoting college students’ academic success and improving education quality.

As one of the crucial components of students’ learning motivation (Han and Lu, 2018), achievement motivation is the driving force behind an individual’s efforts to put energy into what he or she perceives to be valuable and meaningful to achieve a desired outcome (Story et al., 2009). It can be considered as achievement motivation when an individual’s behavior involves “competing at a standard of excellence” (Brunstein and Heckhausen, 2018). Students’ achievement motivation ensures the continuity of learning activities, achieving academic excellence and desired goals (Sopiah, 2021). Based on the concept of achievement motivation, academic achievement motivation refers to the mental perceptions or intentions that students carry out regarding their academic achievement, a cognitive structure by which students perceive success or failure and determine their behavior (Elliot and Church, 1997). Related research also suggests that motivation is one variable that significantly predicts learning engagement (Xiong et al., 2015).

Therefore, it is worthwhile to investigate the internal influence mechanism of college students’ online game addiction on their reduced academic achievement motivation and the role of learning engagement, which is also an issue that cannot be ignored in higher education research. The present study explored the relationship between online game addiction, learning engagement, and reduced academic achievement motivation among college students by establishing a structural equation model (SEM) to shed light on the problem of online game addiction among college students.

## 2. Research model and hypotheses

### 2.1. Research model

**Figure 1:**
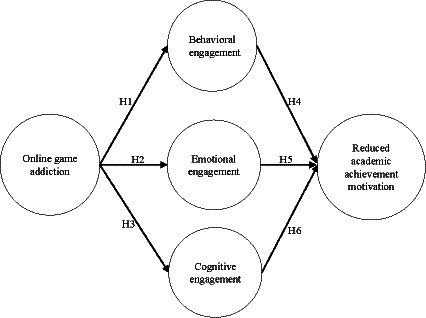
The research model.

Previous research usually regarded learning engagement as a variable of one or two dimensions, and scholars tend to favor the dimension of behavioral engagement. However, other ignored dimensions are inseparable parts of learning engagement (Dincer et al., 2019). In a multi-dimensional model, the mutual terms of each dimension form a single composite structure. Therefore, the present study took the structure proposed by Fredricks et al. (2004) as a reference, divided learning engagement into behavioral, emotional, and cognitive dimensions as mediating variables, and explored the relationship between online game addiction, learning engagement, and reduced academic achievement motivation. The research frame diagram is shown in Figure 1.

### 2.2. Research questions

#### 2.2.1. The relationship between online game addiction and learning engagement

Learning engagement has been viewed as a multidimensional concept in previous studies. Finn (1989) proposed the participation-identification model to make pioneering progress in learning engagement study. Schaufeli et al. (2002) suggested that learning engagement was an active, fulfilling mental state associated with learning. Chapman (2002) pointed out affective, behavioral, and cognitive criteria for assessing students’ learning engagement based on previous research. Fredricks et al. (2004) systematically outlined learning engagement as an integration of behavioral, emotional, and cognitive engagement. The updated International Classification of Diseases [World Health Organization (WHO), 2018a,b] specifies several diagnostic criteria for gaming addiction, including the abandonment of other activities, the loss of interest in other previous hobbies, and the loss or potential loss of work and social interaction because of gaming. Past studies have shown the adverse effects of excessive Internet usage on students’ learning. Short video addiction negatively affects intrinsic and extrinsic learning motivation (Ye et al., 2022). Students’ cell phone addiction negatively affects academic commitment, academic performance, and relationship facilitation, all of which negatively affect their academic achievement (Tian et al., 2021). The amount of time spent surfing the Internet and playing games has been identified to negatively affect students’ cognitive ability (Pan et al., 2022). College students’ cell phone addiction, mainly reflected in cell phone social addiction and game entertainment addiction, has also been noted to impact learning engagement; specifically, the higher the level of addiction, the lower the learning engagement (Qi et al., 2020). Gao et al. (2021) also showed that cell phone addiction among college students could negatively affect their learning engagement. Choi (2019) showed that excessive use of cell phones might contribute to smartphone addiction, which also affects students’ learning engagement. Accordingly, the following three research hypotheses were proposed.

*H1*: Online game addiction negatively affects behavioral engagement.

*H2*: Online game addiction negatively affects emotional engagement.

*H3*: Online game addiction negatively affects cognitive engagement.

#### 2.2.2. The relationship between learning engagement and reduced academic achievement motivation

Achievement motivation is people’s pursuit of maximizing individual value, which embodies an innate drive, including the need for achievement, and can be divided into two parts: the intention to succeed and the intention to avoid failure (McClelland et al., 1976). On this basis, Weiner (1985) proposed the attributional theory of achievement motivation, suggesting that individuals’ personality differences, as well as the experience of success and failure, could influence their achievement attributions and that an individual’s previous achievement attributions would affect his or her expectations and emotions for the subsequent achievement behavior while expectations and emotions could guide motivated behavior. Birch and Ladd (1997) indicated that behavioral engagement involved positive behavioral attitudes such as hard work, persistence, concentration, willingness to ask questions, and active participation in class discussions to complete class assignments. Students’ attitudes toward learning are positively related to achievement motivation (Bakar et al., 2010). Emotional engagement involves students’ sense of identity with their peers, teachers, and the school environment (Stipek, 2002). Students’ perceptions of the school environment influence their achievement motivation (Wang and Eccles, 2013). Cognitive engagement encompasses the ability to use cognitive strategies, self-regulation, investment in learning, and reflective thinking (Khan et al., 2017). Learning independence and problem-solving abilities predict student motivation (Saeid and Eslaminejad, 2017). Hu et al. (2021) indicated that cognitive engagement had the most significant effect on students’ academic achievement among the learning engagement dimensions, and that emotional engagement was also an important factor influencing students’ academic achievement. Therefore, the following three research hypotheses were proposed:

*H4*: Behavioral engagement significantly and negatively affects the reduced academic achievement motivation.

*H5*: Emotional engagement significantly and negatively affects the reduced academic achievement motivation.

*H6*: Cognitive engagement significantly and negatively affects the reduced academic achievement motivation.

#### 2.2.3. The relationship between online game addiction, learning engagement, and reduced academic achievement motivation

Past studies have demonstrated the relationship between online game addiction and students’ achievement motivation. For example, a significant negative correlation between social network addiction and students’ motivation to progress has been reported (Haji Anzehai, 2020), and a significant negative correlation between Internet addiction and students’ achievement motivation has been reported (Cao et al., 2008). Students addicted to online games generally have lower academic achievement motivation because they lack precise academic planning and motivation (Chen and Gu, 2019). Yayman and Bilgin (2020) pointed out a correlation between social media addiction and online game addiction. Accordingly, there might be a negative correlation between online game addiction and academic achievement motivation among college students.

Students addicted to online games generally have lower motivation for academic achievement because they lack precise academic planning and learning motivation (Chen and Gu, 2019). Similarly, Haji Anzehai (2020) reported a significant negative correlation between social network addiction and students’ motivation to progress.

Learning engagement is often explored as a mediating variable in education research. Zhang et al. (2018) found that learning engagement was an essential mediator of the negative effect of internet addiction on academic achievement in late adolescence and is a key factor in the decline in academic achievement due to students’ internet addiction. Li et al. (2019) noted that college students’ social networking site addiction significantly negatively affected their learning engagement, and learning engagement mediated the relationship between social networking addiction and academic achievement. Accordingly, the following research hypothesis was proposed.

*H7*: Learning engagement mediates the relationship between online game addiction and reduced academic achievement motivation.

## 3. Research methodology and design

### 3.1. Survey implementation

The present study employed the Questionnaire Star application for online questionnaire distribution. Convenience sampling was adopted to recruit Chinese college students to participate voluntarily. The data were collected from October 2021 to January 2022 from a higher vocational college in Shandong province, China. Participants were first-and second-year students. According to Shumacker and Lomax (2016), the number of participants in SEM studies should be approximately between 100 and 500 or more. In the present study, 500 questionnaires were returned, and 443 were valid after excluding invalid responses. The mean age of the participants was 18.77 years. There were 157 male students, accounting for 35.4% of the total sample, and 286 female students, accounting for 64.6%.

### 3.2. Measurement instruments

The present empirical study employed quantitative research methods by collecting questionnaires for data analysis. The items of questionnaires were adapted from research findings based on corresponding theories and were reviewed by experts to confirm the content validity of the instruments. The distributed questionnaire was a Likert 5-point scale (1 for *strongly disagree*, 2 for *disagree*, 3 for *average*, 4 for *agree*, and 5 for *strongly agree*). After the questionnaire was collected, item analysis was conducted first, followed by reliability and validity analysis of the questionnaire constructs using SPSS23 to test whether the scale met the criteria. Finally, research model validation was conducted.

#### 3.2.1. Online game addiction

In the present study, online game addiction referred to the addictive behavior of college students in online games, including mobile games and online games. The present study adopted a game addiction scale compiled by Wu et al. (2021) and adapted the items based on the definition of online game addiction. The adapted scale had 10 items. Two examples of the adapted items in the scale were: “I will put down what should be done and spend my time playing online games” and “My excitement or expectation of playing an online game is far better than other interpersonal interactions.”

#### 3.2.2. Learning engagement

In the present study, learning engagement included students’ academic engagement in three dimensions: behavioral, emotional, and cognitive. The learning engagement scale compiled by Luan et al. (2020) was adapted based on its definition. The adapted scale had 26 questions in three dimensions: behavioral, emotional, and cognitive engagement. Two examples of the adapted items in the scale are: “I like to actively explore unfamiliar things when I am doing my homework” and “I will remind myself to double-check the places where I tend to make mistakes in my homework.”

#### 3.2.3. Reduced academic achievement motivation

Reduced academic achievement motivation in the present study refers to the reduction in college students’ intrinsic tendency to enjoy challenges and achieve academic goals and academic success. The achievement motivation scale developed by Ye et al. (2020) was adapted to measure reduced academic achievement motivation. The adapted scale had 10 items. Two examples of the adapted items in the scale are: “Since playing online games, I do not believe that the effectiveness of learning is up to me, but that it depends on other people or the environment” and “Since I often play online games, I am satisfied with my current academic performance or achievement and do not seek higher academic challenges.”

## 4. Results and discussion

### 4.1. Internal validity analysis of the measurement instruments

In the present study, item analysis was conducted using first-order confirmatory factor analysis (CFA), which can reflect the degree of measured variables’ performance within a smaller construct (Hafiz and Shaari, 2013). The first-order CFA is based on the streamlined model and the principle of independence of residuals. According to Hair et al. (2010) and Kenny et al. (2015), it is recommended that the value of *χ*^2^/*df* in the model fitness indices should be less than 5; the root mean square error of approximation (RMSEA) value should be greater than 0.100; the values of the goodness of fit index (GFI) and adjusted goodness of fit index (AGFI) should not be lower than 0.800; the factor loading (FL) values of the constructs should also be greater than 0.500. Based on the criteria above, the items measuring the online game addiction construct were reduced from 10 to seven; the items measuring the behavioral engagement construct were reduced from nine to six; the items measuring the emotional engagement construct were reduced from nine to six; the items measuring the cognitive engagement construct were reduced from eight to six; and the items measuring the reduced academic achievement motivation construct was reduced from 10 to six, as shown in Table 1.

**Table 1:** First-order confirmatory factor analysis.

Model fitness	Threshold value	Online game addiction	Behavioral engagement	Emotional engagement	Cognitive engagement	Reduced academic achievement motivation
*χ* ^2^	–	39.900	29.200	31.600	36.9	15
*df*	–	14	9	9	9	9
*χ*^2^/*df*	<5	2.850	3.324	3.511	4.100	1.667
RMSEA	<0.100	0.065	0.071	0.075	0.084	0.039
GFI	>0.800	0.975	0.977	0.977	0.973	0.989
AGFI	>0.800	0.949	0.947	0.947	0.937	0.974

### 4.2. Construct reliability and validity analysis

In order to determine the internal consistency of the constructs, the reliability of the questionnaire was tested using Cronbach’ s *α* value. According to Hair et al. (2010), a Cronbach’ s *α* value greater than 0.700 indicates an excellent internal consistency among the items, and the constructs’ composite reliability (CR) values should exceed 0.700 to meet the criteria. In the present study, the Cronbach’ s α values for the constructs ranged from 0.911 to 0.960, and the CR values ranged from 0.913 to 0.916, which met the criteria, as shown in Table 2.

**Table 2:** Construct reliability and validity of constructs.

Variables	*M*	*SD*	*α*	FL	CR	AVE
Online game addiction	1.828	0.900	0.911	0.700–0.841	0.913	0.600
Behavioral engagement	4.160	0.879	0.904	0.526–0.903	0.914	0.646
Emotional engagement	3.869	0.904	0.955	0.784–0.910	0.956	0.783
Cognitive engagement	3.891	0.877	0.960	0.779–0.932	0.961	0.805
Reduced academic achievement motivation	2.248	1.080	0.954	0.704–0.920	0.950	0.759

*M* = mean; SD = standard deviation; *α* = Cronbach’ s *α*; FL = factor loading; CR = composite reliability; AVE = average variance extracted.

In the present study, convergent validity was confirmed by two types of indicators, FL and average variance extracted (AVE). According to Hair et al. (2011), an FL value should be greater than 0.500, and items with an FL value less than 0.500 should be removed; and AVE values should be greater than 0.500. In the present study, the FL values of the constructs ranged from 0.526 to 0.932, and the AVE values ranged from 0.600 to 0.805; all dimensions met the recommended criteria, as shown in Table 2.

According to Awang (2015) and Hair et al. (2011), the square root of the AVE of each construct (latent variable) should be greater than its correlation coefficient values with other constructs to indicate the ideal discriminant validity. The results of the present study showed that the three constructs of online game addiction, learning engagement, and reduced academic achievement motivation had good discriminant validity in the present study, as shown in Table 3.

**Table 3:** Discriminant validity analysis.

Variables	1	2	3	4	5
Online game addiction	(0.775)				
Behavioral engagement	−0.402	(0.804)			
Emotional engagement	−0.352	0.696	(0.885)		
Cognitive engagement	−0.288	0.601	0.787	(0.897)	
Reduced academic achievement motivation	0.295	−0.497	−0.528	−0.503	(0.971)

The square roots of AVE values are in parentheses, and the other values are Pearson correlation coefficients.

### 4.3. Correlation analysis

Pearson’s correlation coefficient is usually used to determine the closeness of the relationship between variables. A correlation coefficient greater than 0.8 indicates a high correlation between variables; a correlation coefficient between 0.3 and 0.8 indicates a moderate correlation between variables; while a correlation of less than 0.3 indicates a low correlation. Table 4 shows the Correlation Analysis results. Online game addiction was moderately negatively correlated with behavioral engagement (*r* = −0.402, *p* < 0.001), moderately negatively correlated with emotional engagement (*r* = −0.352, *p* < 0.001), slightly negatively correlated with cognitive engagement (*r* = −0.288, *p* < 0.001), and slightly positively correlated with reduced academic achievement motivation (*r* = 0.295, *p* < 0.001). Behavioral engagement was moderately positively correlated with emotional engagement (*r* = 0.696, *p* < 0.001), moderately positively correlated with cognitive engagement (*r* = 0.601, *p* < 0.001), and moderately negatively correlated with reduced academic achievement motivation (*r* = −0.497, *p* < 0.001). Emotional engagement was moderately positively correlated with cognitive engagement (*r* = 0.787, *p* < 0.001) and moderately negatively correlated with reduced academic achievement motivation (*r* = −0.528, *p* < 0.001). Cognitive engagement was moderately negatively correlated with reduced motivation for academic achievement (*r* = −0.528, *p* < 0.001).

**Table 4:** Correlation analysis.

Variables	1	2	3	4	5
1. Online game addiction	1				
2. Behavioral engagement	−0.402***	1			
3. Emotional engagement	−0.352***	0.696***	1		
4. Cognitive engagement	−0.288***	0.601***	0.787***	1	
5. Reduced academic achievement motivation	0.295***	−0.497***	−0.528***	−0.503***	1

### 4.4. Analysis of fitness of the measurement model

According to Hair et al. (2010) and Abedi et al. (2015), the following criteria should be met in the analysis for measurement model fitness: the ratio of chi-squared and degree of freedom (*χ*^2^/*df*) should be less than 5; the root mean square error of approximation (RMSEA) should not exceed 0.100; the goodness of fit index (GFI), adjusted goodness of fit index (AGFI), normed fit index (NFI), non-normed fit index (NNFI), comparative fit index (CFI), incremental fit index (IFI) and relative fit index (RFI) should be higher than 0.800; and the parsimonious normed fit index (PNFI) and the parsimonious fitness of fit index (PGFI) should be higher than 0.500. The model fitness indices in the present study were *χ*^2^ = 1434.8, *df* = 428, *χ*^2^/*df* = 3.352, RMSEA = 0.073, GFI = 0.837, AGFI = 0.811, NFI = 0.899, NNFI = 0.920, CFI = 0.927, IFI = 0.927, RFI = 0.890, PNFI = 0.827, and PGFI = 0.722. The results were in accordance with the criteria, indicating a good fitness of the model in the present study (Table 5).

**Table 5:** Direct effects analysis.

Path	*β*
Online game addiction→Behavioral engagement	−0.486***
Online game addiction→Emotional engagement	−0.430***
Online game addiction→Cognitive engagement	−0.370 ***
Online game addiction→Reduced academic achievement motivation	0.19**
Behavioral engagement→Reduced academic achievement motivation	−0.238***
Emotional engagement→Reduced academic achievement motivation	−0.221**
Cognitive engagement→Reduced academic achievement motivation	−0.265***

**p < 0.01, ***p < 0.001.

### 4.5. Validation of the research model

**Figure 2:**
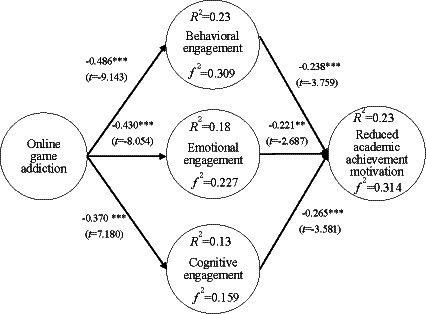
Validation of the research model. ***p < 0.001.

Online game addiction had a negative effect on behavioral engagement (*β* = −0.486; *t* = −9.143; *p* < 0.001). Online game addiction had a negative effect on emotional engagement (*β* = −0.430; *t* = −8.054; *p* < 0.001). Online game addiction had a negative effect on cognitive engagement (*β* = −0.370; *t* = −7.180; *p* < 0.001). Online game addiction had a positive effect on reduced academic achievement motivation (*β* = 0.19; *t* = −2.776; *p* < 0.01). Behavioral engagement had a negative effect on reduced academic achievement motivation (*β* = −0.238; *t* = −3.759; *p* < 0.001). Emotional engagement had a negative effect on reduced academic achievement motivation (*β* = −0.221; *t* = −2.687; *p* < 0.01), and cognitive engagement had a negative effect on reduced academic achievement motivation (*β* = −0.265; *t* = −3.581; *p* < 0.01), as shown in Figure 2 Table 6.

**Table 6:** Indirect effects analysis.

Path	*β*	95% CI
Online game addiction→Behavioral engagement→Reduced academic achievement motivation	0.230**	[0.150, 0.300]
Online game addiction→Emotional engagement→Reduced academic achievement motivation	0.209**	[0.130, 0.290]
Online game addiction→Cognitive engagement→Reduced academic achievement motivation	0.170**	[0.100, 0.250]

**p < 0.01.

Cohen’ s *f* ^2^ is an uncommon but valuable standardized effect size measure that can be used to assess the size of local effects (Selya et al., 2012). When *f*^2^ reaches 0.02 it represents a small effect size, 0.150 represents a medium effect size, and 0.350 represents a high effect size (Hair et al., 2014). The explanatory power of online game addiction on behavioral engagement was 23.6%, and *f*^2^ was 0.309. The explanatory power of online game addiction on emotional engagement was 18.5%, and *f*^2^ was 0.227. The explanatory power of online game addiction on cognitive engagement was 13.7%, and *f*^2^ was 0.159. The explanatory power of behavioral, emotional, and cognitive engagement on reduced academic achievement motivation was 23.9%, and *f*^2^ was 0.314. Figure 2 illustrates the above findings.

### 4.6. Indirect effects analysis

Scholars are often interested in whether variables mediate the association between predicting and outcome variables. Therefore, mediating variables can partially or entirely explain the association (Hwang et al., 2019). In research fields such as psychology and behavior, where the research situation is often more complex, multiple mediating variables are often required to clearly explain the effects of the independent variables on the dependent variables (MacKinnon, 2012). Scientific quantitative research requires tests of confidence interval (CI; Thompson, 2002), and the standard value of the test numbers is often determined by 95% CI (Altman and Bland, 2011). CI value not containing 0 indicates the statistical significance of the analysis results (Nakagawa and Cuthill, 2007). According to the statistical results shown in Table 4, behavioral engagement significantly positively mediated the relationship between online game addiction and reduced academic achievement motivation with a path coefficient of 0.230 and 95% CI ranging from 0.150 to 0.300 (excluding 0), *p* < 0.01; emotional engagement positively mediated the relationship between online game addiction and reduced academic achievement motivation with a path coefficient of 0.209, 95% CI ranging from 0.130 to 0.292 (excluding 0), *p* < 0.01; cognitive engagement positively mediated the relationship between online game addiction and reduced academic achievement motivation with a path coefficient of 0.170, 95% CI ranging from 0.100 to 0.250 (excluding 0), *p* < 0.01, as shown in Table 6.

### 4.7. Discussion

#### 4.7.1. Analysis of the relationship between online game addiction and learning engagement

Online game addiction is often negatively associated with students’ learning. For example, the problematic use of short videos was reported as negatively affecting students’ behavioral engagement, while behavioral engagement positively affected students’ emotional and cognitive engagement (Ye et al., 2023). Meral (2019) highlighted that students’ learning attitudes and academic performance had a negative relationship with students’ addiction to online games. Demir and Kutlu (2018) found that online game addiction negatively affects students’ learning motivation. As the level of students’ game addiction increased, the level of their communication skills decreased (Kanat, 2019). Furthermore, Tsai et al. (2020) pointed out a negative correlation between online game addiction and peer relationships as well as students’ learning attitudes. According to the results of the research model validation, it can be observed that: online game addiction negatively affected behavioral engagement, emotional engagement, and cognitive engagement. Therefore, it can be stated that online game addiction had significant and negative effects on all dimensions of learning engagement.

Online game addiction in the present study included aspects of computer game addiction and mobile phone game addiction. The results of the present study are consistent with the findings of Gao et al. (2021), Choi (2019), and Qi et al. (2020), who pointed out that college students’ addiction to cell phones negatively affected their learning engagement.

#### 4.7.2. Analysis of the relationship between learning engagement and reduced academic achievement motivation

For technology education in higher education, students’ intrinsic motivation for academic study predicts their learning engagement (Dunn and Kennedy, 2019). In addition, learning engagement is positively correlated with academic achievement (Fredricks and McColskey, 2012). Based on the research model validation results, behavioral, emotional, and cognitive engagement all negatively affected reduced academic achievement motivation. The findings are consistent with Hu et al.’s (2021) study which pointed out that cognitive engagement in the learning engagement dimension had the most significant effect on students’ academic achievement, and that emotional engagement was also an essential factor influencing students’ academic achievement. Lau et al. (2008) showed that achievement motivation positively predicted cognitive engagement in the learning engagement dimension. Mih et al. (2015) noted that achievement motivation positively predicted behavioral and emotional engagement in the learning engagement dimension. The present study supported the above discussion by confirming the association between learning engagement and reduced academic achievement motivation.

#### 4.7.3. Analysis of the mediating role of learning engagement

According to the indirect effects analysis results of the present study, learning engagement negatively mediated the relationship between online game addiction and reduced academic achievement motivation. The findings support Haji Anzehai’s (2020) conclusion that social network addiction negatively correlated with students’ motivation to progress (Haji Anzehai, 2020). It is also consistent with the findings of Chen and Gu (2019) that students addicted to online games generally had lower academic achievement motivation due to a lack of precise academic planning and motivation. Cao et al. (2008) found a significant negative correlation between Internet addiction and students’ achievement motivation. Similarly, Zhang et al. (2018) explored the intrinsic influencing mechanism of students’ Internet addiction on academic achievement decline in their late adolescence by identifying learning engagement as the important mediating variable. Li et al. (2019) proposed that social networking site addiction among college students significantly negatively affected learning engagement and that learning engagement mediated the relationship between social network addiction and students’ academic achievement. The present study findings also support the discussion above.

## 5. Conclusion and suggestions

### 5.1. Conclusion

Currently, the problem of online game addiction among college students is increasing. The relationship between online game addiction, learning engagement, and reduced academic achievement motivation still needs to be explored. The present study explored the relationships between the three aforementioned variables by performing SEM. The results of the study indicated that: (1) online game addiction negatively affected behavioral engagement; (2) online game addiction negatively affected emotional engagement; (3) online game addiction negatively affected cognitive engagement; (4) behavioral engagement negatively affected reduced academic achievement motivation; (5) emotional engagement negatively affected reduced academic achievement motivation; (6) cognitive behavioral engagement negatively affected reduced academic achievement motivation; (7) learning engagement mediated the relationship between online game addiction and reduced academic achievement motivation.

According to the research results, when college students are addicted to online games, their learning engagement can be affected, which may decrease their behavioral, emotional, and cognitive engagement; their academic achievement motivation may be further reduced and affect their academic success or even prevent them from completing their studies. The mediating role of learning engagement between online game addiction and reduced academic achievement motivation indicates that reduced academic achievement motivation influenced by online game addiction could be prevented or weakened by enhancing learning engagement.

### 5.2. Suggestions

Universities and families play a crucial role in preventing online game addiction among college students. One of the main reasons college students play online games may be that they lack an understanding of other leisure methods and can only relieve their psychological pressure through online games (Fan and Gai, 2022). Therefore, universities should enrich college students’ after-school leisure life and help them cultivate healthy hobbies and interests. Besides, a harmonious parent–child relationship positively affects children’s learning engagement (Shao and Kang, 2022). Parents’ stricter demands may aggravate children’s game addiction (Baturay and Toker, 2019). Therefore, parents should assume a proper perspective on the rationality of gaming and adopt the right approach to guide their children.

One key factor influencing the quality of higher education is students’ learning engagement. The integration of educational information technology has disrupted traditional teaching methods. This trend has accelerated in the context of COVID-19. College students’ growth mindset can impact their learning engagement through the role of the perceived COVID-19 event strength and perceived stress (Zhao et al., 2021). Moreover, students’ self-regulated learning and social presence positively affect their learning engagement in online contexts (Miao and Ma, 2022). Students’ liking of the teacher positively affects their learning engagement (Lu et al., 2022). Their perceived teacher support also positively affects their learning engagement (An et al., 2022). Hence, educators should focus on teacher support and care in the teaching and learning process.

Students’ motivation for academic achievement can often be influenced by active interventions. Cheng et al. (2022) noted that the cumulative process of students gaining successful experiences contributed to an increased sense of self-efficacy, motivating them to learn. Zhou (2009) illustrated that cooperative learning motivated students’ academic achievement. In addition, Hong J. C. et al. (2021) showed that poor parent–child relationships (such as the behavior of “mama’ s boy” in adults) had a negative impact on students’ academic achievement motivation, and they concluded that cell phone addiction was more pronounced among students with low academic achievement motivation. Hence, enhancing students’ academic achievement motivation also requires family support.

### 5.3. Research limitations and suggestions for future research

Most of the past studies on the impact of online game addiction on academics have used quantitative research as the research method. The qualitative research approach regarding students’ online game addiction should not be neglected. By collecting objective factual materials in the form of qualitative research such as interviews a greater understanding of students’ actual views on games and the psychological factors of addiction can be achieved. Therefore, future studies could introduce more qualitative research to study online game addiction.

To pay attention to the problem of students’ online game addiction, universities and families should not wait until they become addicted and try to remedy it, but should start to prevent it before it gets to that stage. In terms of developing students’ personal psychological qualities, students’ sensation-seeking and loneliness can significantly affect their tendency to become addicted to online games (Batmaz and Çelik, 2021). Adolescents’ pain intolerance problems can also contribute to Internet overuse (Gu, 2022). Emotion-regulation methods affect the emotional experience and play a vital role in Internet addiction (Liang et al., 2021). In this regard, it is necessary to pay attention to students’ mental health status and to guide them to establish correct values and pursue goals through psychological guidance and other means.

In addition to individual factors, different parenting can considerably impact adolescents. Adolescents who tend to experience more developmental assets are less likely to develop IGD (Xiang et al., 2022a), and external resources can facilitate the development of internal resources, discouraging adolescents from engaging in IGD (Xiang et al., 2022b). Relevant research indicates that the most critical factor in adolescents’ game addiction tendency comes from society or their parents rather than being the adolescents’ fault (Choi et al., 2018). Adolescents who tend to be addicted to online games may have discordant parent–child relationships (Eliseeva and Krieger, 2021). Better father-child and mother–child relationships predict lower initial levels of Internet addiction in adolescents (Shek et al., 2019). Family-based approaches such as improved parent–child relationships and increased communication and understanding among family members can be a direction for adolescent Internet addiction prevention (Yu and Shek, 2013).

At the school level, a close teacher-student relationship is one of the main factors influencing students’ psychological state. Students’ participation in and control over the teaching and learning process as well as their closeness to teachers can increase their satisfaction and thus enhance their learning-related well-being (Yang J. et al., 2021). More school resources can lead to higher adolescent self-control, attenuating students’ online gaming disorders (Xiang et al., 2022c).

## Data availability statement

The raw data supporting the conclusions of this article will be made available by the authors, without undue reservation.

## Ethics statement

Ethical review and approval was not required for the study on human participants in accordance with the local legislation and institutional requirements. Written informed consent for participation was not required for this study in accordance with the national legislation and the institutional requirements. Written informed consent was not obtained from the individual(s) for the publication of any potentially identifiable images or data included in this article.

## Author contributions

R-QS, and J-HY: concept and design and drafting of the manuscript. R-QS, and J-HY: acquisition of data and statistical analysis. G-FS, and J-HY: critical revision of the manuscript. All authors contributed to the article and approved the submitted version.

## Funding

This work was supported by Beijing Normal University First-Class Discipline Cultivation Project for Educational Science (Grant number: YLXKPY-XSDW202211). The Project Name is “Research on Theoretical Innovation and Institutional System of Promoting the Modernization of Vocational Education with Modern Chinese Characteristics”.

## Conflict of interest

The authors declare that the research was conducted in the absence of any commercial or financial relationships that could be construed as a potential conflict of interest.

## Publisher’s note

All claims expressed in this article are solely those of the authors and do not necessarily represent those of their affiliated organizations, or those of the publisher, the editors and the reviewers. Any product that may be evaluated in this article, or claim that may be made by its manufacturer, is not guaranteed or endorsed by the publisher.
